# Analysis of genomic and characterization features of *Luteolibacter soli* sp. nov., isolated from soil

**DOI:** 10.3389/fmicb.2024.1483195

**Published:** 2024-09-13

**Authors:** Jing An, Xiaoqi Xuan, Yanan Wang, Linwei Wu, Jizhong Zhou, Dashuai Mu

**Affiliations:** ^1^Marine College, Shandong University, Weihai, China; ^2^Institute of Ecology, Key Laboratory for Earth Surface Processes of the Ministry of Education, College of Urban and Environmental Sciences, Peking University, Beijing, China; ^3^Department of Microbiology and Plant Biology, University of Oklahoma, Norman, OK, United States; ^4^Institute for Environmental Genomics, University of Oklahoma, Norman, OK, United States; ^5^State Key Joint Laboratory of Environment Simulation and Pollution Control, School of Environment, Tsinghua University, Beijing, China; ^6^School of Civil Engineering and Environmental Sciences, University of Oklahoma, Norman, OK, United States; ^7^School Key Laboratory of Microbial Technology, Shandong University, Qingdao, China; ^8^Weihai Research Institute of Industrial Technology of Shandong University, Weihai, China

**Keywords:** *Luteolibacter*, 16S rRNA gene, genome, phylogenetic, climate change

## Abstract

The strain designated as Y139^T^ is a novel Gram-stain-negative, aerobic, and non-motile bacterium, was isolated from a soil sample in McClain County, Oklahoma, United States. The cells of strain Y139^T^ were a rod-shaped, with the width of 0.4–0.7 μm and the length of 1.5–2.0 μm . Growth occurred at 20–37°C (optimum, 30°C), pH 5.5–9.5 (optimum, pH 7.0), and 0–1.0% NaCl (w/v) (optimum, 0%). The polar lipid profiles included phosphatidylethanolamine, phosphatidylglycerol, diphosphatidylglycerol, phosphatidyldimethylethanolamine, and an unidentified lipid. The major fatty acids included C_16:0_, iso-C_14:0_, and C_16:1_*ω*9*c*. Menaquinone-9 (MK-9) was recognized as the only respiratory quinone. Strain Y139^T^ showed the highest 16S rRNA gene sequence similarity to *Luteolibacter flavescens* MCCC 1K03193^T^ (98.3%). Phylogenetic analysis positioned it within the genus *Luteolibacter*. The draft genome of strain Y139^T^ consisted of 7,106,054 bp, and contained 5,715 open reading frames (ORFs), including 5,656 coding sequences (CDSs) and 59 RNA genes. The genomic DNA G + C content was found to be 62.5%. Comparing strain Y139^T^ with *L. flavescens* MCCC 1K03193^T^ and *Luteolibacter arcticus* CCTCC AB 2014275^T^, the average nucleotide identity (ANI) values were 80.6 and 82.1%, respectively. Following phylogenetic, physiological, biochemical, and chemotaxonomic analyses, a novel species within the genus *Luteolibacter*, designated as *Luteolibacter soli* sp. nov., was proposed for strain Y139^T^, which was also assigned as the type strain (=KCTC 92644^T^ = MCCC 1H01451^T^). Further analysis of core genes across 9 *Luteolibacter* species uncovered significant genomic divergence, particularly in those related to cofactor, vitamin, and energy metabolism. Analysis of biogeographic distribution suggested that lake and soil were the main habitats for the genus *Luteolibacter*. Additionally, the genus *Luteolibacter* was sensitive to climate warming and precipitation.

## 1 Introduction

The genus *Luteolibacter* is classified within the phylum *Verrucomicrobiota*. *Luteolibacter pohnpeiensis* is the type species of the genus *Luteolibacter* which was first described in 2008 by Yoon et al. (2008). This genus includes 11 validly named species listed in LPSN.^1^ The genus *Luteolibacter* has been found in various environments, including marine environments (Zhang et al., 2017; Xie et al., 2022; Zhou et al., 2023), tundra soil (Jiang et al., 2012; Kim et al., 2015), activated sludge (Park et al., 2013), soil (Pascual et al., 2017; Dahal et al., 2021), skin of Anderson’s salamander (Busse et al., 2021) and *Hirudo medicinalis* (Glaeser et al., 2012). The major respiratory quinone present in *Luteolibacter* species is Menaquinone-9 (MK-9). The genomic DNA G + C content of the DNA in these species varies from 53.5 to 65.0% (Yoon et al., 2008; Xie et al., 2022).

The significance of global climate change is undeniable, as it has widespread impacts on ecosystems. Microorganisms are crucial in climate change due to their wide distribution, short generation times and larger populations (Zhang and Xu, 2008; Collins, 2011; Thomas et al., 2012). In terrestrial ecosystems, soil serves as the largest carbon sink, small changes in soil microorganisms can cause significant changes in climate feedback. The phylum *Verrucomicrobiota* is commonly found in soil environments, especially in grasslands and soil horizons. This suggests that members of this phylum may play significant roles in the soil environment (Kielak et al., 2009; Bergmann et al., 2011). The phylum *Verrucomicrobiota* has a significant impact on the global biogeochemical cycling process, particularly in terms of their contribution to nutrient cycling (Banerjee et al., 2016). Despite the acknowledged significance of *Verrucomicrobiota* in the environment, research on their responses to climate change remains limited.

The composition and functionality of microbial communities may shift due to climate change (Zhou et al., 2012; Xu et al., 2013; Guo et al., 2018; Wu et al., 2022). The phylum *Verrucomicrobiota* has been revealed to be sensitive to climate warming and precipitation. This sensitivity could have several implications. (a) Distribution: changes in temperature and precipitation patterns could affect the geographic distribution (Oliverio et al., 2017); (b) Abundance: population sizes might fluctuate in response to climatic variations, a rise in temperature triggered a significant drop in both species diversity and relative abundance. Increased precipitation can enhance the relative abundance, indicating their favorable survival in environments with higher moisture content (Zhang et al., 2018; Zhao et al., 2024); (c) Activity: metabolic rates and ecological functions could be impacted by changing environmental conditions. Our research focused on an aerobic soil isolate, recognized as a novel species of genus *Luteolibacter* from the phylum *Verrucomicrobiota*. Enhancing the genus *Luteolibacter* will support future research on microbial responses to climate variability.

## 2 Materials and methods

### 2.1 Study site and sampling

The Kessler Atmospheric and Ecological Field Station (KAEFS) is located in McClain County, Oklahoma, in the US Great Plains (34° 59′ N, 97° 31′ W) (Guo et al., 2018). The site is mainly composed of C3 and C4 herbaceous plants (Xu et al., 2013). From 1948 to 1999, the average temperature in January was 3.3°C and that in July was 28.1°C, with an annual average of 16.3°C. Precipitation ranged from 82 to 240 mm, with an average annual total of 914 mm. The soils are classified as a Pulaski-Port Keokuk complex, which is characterized by a loamy texture (Li et al., 2013). In July 2009, a field experiment was built. A blocked split-plot experiment was built to analyze the impact of three climate factors on ecosystems: warming, precipitation, and clipping. The warming experimental plots were heated continuously to maintain a temperature of 3°C above ambient. Precipitation adjustment consisted of setting the target level to +100% of ambient precipitation to simulate increased rainfall. Also, aboveground biomass was clipped annually during the peak growing season (Guo et al., 2018; Wu et al., 2022).

### 2.2 Amplicon sequencing and data preprocessing

The construction of the 16S rRNA gene (V4 region) library involved a two-step PCR amplification method, followed by Wu et al. (2022). The paired-end sequences obtained were subjected to primer sequence trimming. After trimming, the sequences were merged using FLASH. Merged sequences with ambiguous bases or a length of less than 245 bp for the 16S rRNA gene were not included in the subsequent analysis. High-quality sequences of the 16S rRNA gene that remained were then processed to create amplicon sequence variants (ASVs). This processing step was performed using UNOISE3 (Edgar, 2018).

### 2.3 Bacterial isolation and cultivation

A soil sample was obtained from a grassland site at KAEFS in August 2009. The sampling depth was established at approximately 0–15 cm below the surface, then soil samples were stored at −80°C (Guo et al., 2018). In 2020, 1 g of soil sample was serially diluted to 10^−2^–10^−4^ in sterilized water and 0.1 mL aliquots of each dilution were spread onto the surface of 1/3R2A agar medium. The R2A agar medium contained (g/L): tryptone 0.5, yeast 0.5, casein 0.5, starch 0.5, glucose 0.5, K_2_HPO_4_ 0.3, sodium pyruvate 0.3, and MgSO_4_∙7H_2_O 0.05, agar 15 g, all w/v, pH 7.2. The incubation of the agar plates was carried out aerobically at 28°C for around 10 days. A yellow colony was isolated from the incubated plates and subsequently purified through successive subcultures on R2A agar identified as strain Y139^T^. Pure cultures of the strains were preserved at −80°C supplemented with 15% (v/v) glycerol.

### 2.4 16S rRNA gene sequence analysis

PCR amplification was performed using the universal primers 27F and 1492R (Liu et al., 2014). The ContEst16S algorithm was used to extract the complete sequence from the genome and submit it to the GenBank database (Lee et al., 2017). The 16S rRNA sequences were compared using BLAST algorithms on GenBank^2^ and the EzTaxon-e server^3^ to identify the phylogenetic position of strain Y139^T^ (Kim et al., 2012). The phylogenetic trees were reconstructed in MEGA 11 using neighbor-joining (NJ), maximum-parsimony (MP), and maximum-likelihood (ML) methods (Felsenstein, 1981; Saitou and Nei, 1987; Kannan and Wheeler, 2012; Kumar et al., 2018). The ML tree reconstruction employed the model GTR + G + I. To ascertain the confidence level of the branch nodes, a bootstrap test with 1,000 replicates was performed.

### 2.5 Genomic analyses

Using a bacterial genomic DNA kit (TaKaRa Biotechnology, Japan), the genomic DNA of strain Y139^T^ was extracted and purified, and subsequently sequenced by Beijing Novogene Bioinformatics Technology (Beijing, China) on the NovaSeq 6,000 sequencing platform. Genome sequences of *Luteolibacter flavescens* MCCC 1K03193^T^ (GCA_025950085.1), *Luteolibacter. pohnpeiensis* CCTCC AB 2011006^T^ (GCA_016595435.1) and *Luteolibacter arcticus* CCTCC AB 2014275^T^ (GCA_025950235.1) were downloaded from the NCBI database. Completeness and contamination levels of genomes were evaluated with CheckM (Parks et al., 2015). To annotate the genome information, the NCBI prokaryotic genome annotation pipeline (PGAP) was used. The average amino acid identity (AAI) value was computed with CompareM. The average nucleotide identity (ANI) value was calculated using an online ANI tool^4^ (Figueras et al., 2014). The digital DNA–DNA hybridization (dDDH) values were calculated using the Genome-to- Genome Distance Calculator.^5^ The JTT + CAT parameters were used in FastTree and the LG + F + I + G4 model was used in IQ-Tree to reconstruct phylogenetic trees (Price et al., 2010; Trifinopoulos et al., 2016). For tree support, 1,000 bootstrap replicates were used in both approaches.

### 2.6 Comparative genomics of the genus *Luteolibacter*

Genomes of type species within the genus *Luteolibacter* were obtained from NCBI. The genomes were annotated, and comparative genomics analysis was performed of the genus *Luteolibacter* through three databases: KEGG database^6^ (Kanehisa et al., 2023), RAST database^7^ (Aziz et al., 2008) and Anti-SMASH database^8^ (Blin et al., 2019). To assess genomic diversity and identify orthologous groups among the members of the genus *Luteolibacter*, pan-genome analysis using the bacterial pan-genome analysis (BPGA) tool was performed with default parameters (50% amino acid sequence identity) (Chaudhari et al., 2016).

### 2.7 Physiological and biochemical characteristics

Cells were grown on R2A agar at 30°C for 3 days to investigate the morphological features of strain Y139^T^. A Gram stain kit (Hopebio, Qingdao, China) was used to perform Gram staining. The motility characteristics were assessed by examining gliding motility (Bernardet et al., 2002). Light microscopy (Nikon E600) and scanning electron microscopy (Nova NanoSEM 450; FEI) were used to determine the morphology and size of the cells. The growth conditions for strain Y139^T^ were evaluated across a range of temperatures (0, 4, 10, 15, 20, 25, 28, 30, 33, 37, 40, 42, and 45°C) on R2A agar. To investigate the salt tolerance of cells, they were cultured in R2A medium with NaCl added at concentrations of 0, 0.5, 1, 1.5, 2, 3, 4 and 5% (w/v). The range of pH values (5.5–9.5) was assessed by adding specific buffers into the R2A medium: MES (pH 5.5–6.0), PIPES (pH 6.5–7.0), HEPES (pH 7.5–8.0), Tricine (pH 8.5), and CAPSO (pH 9.0–9.5) at a concentration of 20 mM. Using a reagent kit from bioMérieux, oxidase activity was assessed. Catalase activity was determined by adding 3% (v/v) hydrogen peroxide solution and observing the bubbles formed. Anaerobic growth was evaluated on R2A plates with or without 0.1% (w/v) KNO_3_, incubated in an anaerobic jar (containing 10% H_2_, 10% CO_2_ and 80% N_2_) at 30°C for 14 days. Enzymatic activity toward substrates including alginate, CM-cellulose, starch and lipids (Tweens 20, 40, 60, and 80) were investigated. After incubating for 3 days at 30°C, the sizes of the inhibition zones surrounding the antibiotic-impregnated disks were tested to determine antibiotic susceptibility (Jorgensen and Turnidge, 2015). Subsequently, they were cultured under optimal conditions, using API ZYM, API 20E, API 50CHB (bioMérieux) strips, and Biolog GEN III systems. The physiological and biochemical traits of strain Y139^T^ and experimental strains were analyzed, followed by the manufacturer’s directions. Experimental strains of *L. pohnpeiensis* CCTCC AB 2011006^T^ and *L. arcticus* CCTCC AB 2014275^T^ were acquired from the China Center for Type Culture Collection (CCTCC) (Yoon et al., 2008; Kim et al., 2015), *L. flavescens* MCCC 1K03193^T^ was obtained from Marine Culture Collection of China (MCCC) (Zhang et al., 2017).

### 2.8 Chemotaxonomy

Strain Y139^T^ was cultured for 2 days on R2A at 30°C at a pH of 7.0, then harvested during the exponential growth phase and freeze-dried. Fatty acids, polar lipids, and respiratory quinones were extracted using these preparations. Menaquinone was extracted following the methods described by Minnikin et al. (1984), and then analyzed by high-performance liquid chromatography (HPLC) (Kroppenstedt, 1982). The Microbial Identification System (Sherlock version 4.5; database: TSBA40; MIDI) was used to identify cellular fatty acid methyl esters (FAMEs) (Chou et al., 1990). Additionally, polar lipids were extracted with a chloroform/methanol system and analyzed by two-dimensional thin-layer chromatography (Du et al., 2014).

### 2.9 Biogeographic distribution of genus *Luteolibacter*

The distribution and habitat preferences of *Luteolibacter* globally were assessed using the analytical tools from the Microbe Atlas Project (MAP).^9^ The study was conducted with a rigorous 96% sequence similarity threshold. Microbial community abundance was assessed through MAPseq, a closed reference method for analyzing ribosomal RNA sequences (Matias Rodrigues et al., 2017).

### 2.10 Data analysis

The trends of relative abundances of the phylum *Verrucomicrobiota* and the genus *Luteolibacter* were statistically analyzed using different plots as replicates. The t-test was conducted with the R package stats (v4.4.1, 2024).

## 3 Results and discussion

### 3.1 Phenotypic properties

Yellow colonies formed by strain Y139^T^ were observed during cultivation on R2A agar plates. The width and length of the cells were found to be 0.4–0.7 μm and 1.5–2.0 μm, respectively (Supplementary Figure S1). Strain Y139^T^ exhibited growth at temperatures ranging from 20 to 37°C (optimum 30°C); tolerated NaCl concentrations from 0 to 1.0% (w/v) (optimum 0%); and pH conditions from 5.5 to 9.5 (optimum 7.0). It exhibited the capacity to hydrolyze cellulose and starch, but could not hydrolyze Tweens 20, 40, 60, 80, alginate, or casein. All strains were positive for tryptophan deaminase, arabinose, oxidase and catalase; negative for casein, nitrite reduction and Tween 20. Same as experimental strains, Y139^T^ was positive for alkaline phosphatase, leucine arylamidase, acid phosphatase, naphthol-AS-BI-phosphohydrolase, *β*-galactosidase, esterase lipase (C8) and esterase (C4). Negative for lipase (C14), cystine arylamidase, *α*-chymotrypsin, *β*-glucuronidase, *α*-glucosidase and N-acetyl-*β*-glucosaminidase. Moreover, negative activities were observed for *α*-galactosidase, *β*-glucosidase and *α*-mannosidase of strain Y139^T^. In API 50CHB strips, acids were produced from d-mannose, glycerol, d-glucose, d-fructose, d-sorbitol, amygdalin, esculin ferric citrate, salicin, d-saccharose, d-melibiose, d-lactose, d-maltose, d-trehalose, inulin, d-raffinose, glycogen, gentiobiose, methyl-*α*-d-mannopyranoside, methyl-*α*-d-glucopyranoside, arbutin, d-melezitose and potassium 5-ketogluconate of all strains. Strain Y139^T^ displayed positive results for d-xylose, d-galactose, d-cellobiose, d-turanose and potassium gluconate, distinguishing it from the experimental strains. In the Biolog GEN III systems, all strains were negative for propionic acid, *β*-hydroxy-d, *α*-keto-butyric acid, *α*-hydroxy-butyric acid, d-malic acid, d-aspartic acid, l-butyric acid and *γ*-amino-butyric acid. Unlike the experimental strains, strain Y139^T^ was negative for fusidic acid, *ρ*-hydroxy-phenylacetic acid and l-malic acid. Table 1 outlines the main features of strain Y139^T^ and experimental strains. Strain Y139^T^ displayed resistance to streptomycin (10 μg per disk), chloramphenicol (30 μg per disk), neomycin (30 μg per disk), carbenicillin (100 μg per disk), vancomycin (30 μg per disk), norfloxacin (30 μg per disk), penicillin (10 μg per disk), ampicillin (10 μg per disk), erythromycin (15 μg per disk), tobramycin (10 μg per disk), and exhibited intermediate resistance to kanamycin (30 μg per disk) and gentamycin (10 μg per disk). Conversely, it was susceptible to ofloxacin (5 μg per disk), ceftriaxone (30 μg per disk), cefotaxime sodium (30 μg per disk), polymyxin B (300 μg per disk), tetracycline (30 μg per disk) and rifampin (5 μg per disk).

**Table 1:** Differential characteristics of strain Y139^T^ and experimental strains.

Characteristic	1	2	3	4
Morphology	Short rods	Short rods^a^	Rods^b^	Rods^c^
Isolation source	Grassland soil	Tundra soil^a^	Deep seawater^b^	Driftwood^c^
Pigment	Yellow	Pale yellow^a^	Yellow^b^	Pale yellow^c^
Growth				
Temperature (optimum) (°C)	20–37	4–37^a^	20–35^b^	20–37^c^
NaCl (optimum) (%)	0–1	0–1^a^	0–4^b^	0–6^c^
pH range	5.5–9.5	5.0–9.0^a^	5.5–9.5^b^	6.5–9.0^c^
Hydrolysis of				
Tweens 40 and 60	−	+	+	−
Tween 80	−	+	−	−
starch	+	+	−	+
cellulose	+	−	−	−
API ZYM strips				
valine arylamidase	+	−	+	−
trypsin	+	−	−	+
*α*-galactosidase/ *β*-glucosidase/*α*-mannosidase	−	−	−	+
50CHB strips				
methyl-*β*-d-xylopyranoside	−	+	−	−
l-rhamnose	−	+	+	−
d-lyxose/potassium 2-ketogluconate	−	−	+	−
d-xylose	+	−	+	−
d-galactose/d-cellobiose/d-turanose	+	−	+	+
potassium gluconate	+	+	−	+
Biolog GEN III systems				
Fusidic acid	−	−	+	+
*ρ*-hydroxy-phenylacetic acid	−	−	−	+
l-malic acid	−	+	−	+
d-raffinose/ nalidixic acid	+	−	+	+
l-fucose/ d-fructose-6-PO_4_	+	+	+	−
d-sorbitol/ *α*-keto-glutaric acid/ N-acetyl neuraminic acid	+	+	−	+
d-serine/bromo-succinic acid/stachyose	+	−	−	+
Quinone	MK-9	MK-9, −10 ^a^	MK-9 ^b^	MK-9, −10 ^c^
Genomic DNA G + C content (%)	62.5	62.2 ^a^	60.7 ^b^	55.8 ^c^

Strains: 1, Y139^T^; 2, Luteolibacter arcticus CCTCC AB 2014275^T^; 3 *Luteolibacter flavescens* MCCC 1K03193^T^; 4 *Luteolibacter pohnpeiensis* CCTCC AB 2011006^T^. All data are from this study unless otherwise indicated. +, Positive; −, Negative. All strains were positive for oxidase and catalase; but negative for casein, nitrite reduction, Tween 20 or casein. Data from: ^a^(Kim et al., 2015); ^b^(Zhang et al., 2017); ^c^(Yoon et al., 2008).

### 3.2 Chemotaxonomic features

Consistent with strains of the genus *Luteolibacter*, MK-9 was the only respiratory quinone of strain Y139^T^ (Yoon et al., 2008). C_16:0_, iso-C_14:0,_ and C_16:1_ *ω*9*c* are the major cellular fatty acids that were similar to the profiles of the experimental strains. Unlike strain Y139^T^, C_16:0_ is the main fatty acid of *L. flavescens* MCCC 1K03193^T^. C_17:0_ 3-OH was identified in strain Y139^T^, but it was not found in other strains. The differences in the proportions of some fatty acids are shown in Table 2. Five polar lipids were identified of strain Y139^T^, including phosphatidylglycerol (PG), phosphatidylethanolamine (PE), phosphatidyldimethylethanolamine (PME), diphosphatidylglycerol (DPG), and an unidentified lipid (L). The presence of diphosphatidylglycerol (DPG), phosphatidylglycerol (PG) and phosphatidylethanolamine (PE) were conserved in all strains. Phosphatidyldimethylethanolamine (PME) was found in *L. pohnpeiensis* CCTCC AB 2011006^T^, but not in other experimental strains. Further detailed polar lipids with different specific strains are given in Supplementary Figure S2.

**Table 2:** Major cellular fatty acid comparison of strain Y139^T^ and experimental strains of the genus *Luteolibacter*.

	1	2	3	4
Straight-chain fatty acids				
C_13:0_	0.7	0.9	1.6	tr
C_14:0_	6.6	7.2	**13.9**	7.1
C_16:0_	**13.2**	**16.3**	**19.4**	**27.1**
C_17:0_	2.5	1.7	tr	0.8
C_18:0_	0.8	–	0.8	1.1
Branched fatty acids				
iso-C_14:0_	**33.2**	**34.0**	**27.8**	**34.0**
anteiso-C_15:0_	4.0	4.2	3.9	8.2
iso-C_16:0_	2.1	1.7	0.8	4.3
iso-C_17:0_	tr	tr	–	tr
Unsaturated fatty acids				
C_15:1_ *ω*8*c*	4.6	3.1	1.3	–
C_16:1_ *ω*9*c*	**21.1**	**22.2**	**20.8**	–
C_17:1_ *ω*8*c*	1.1	–	–	tr
Hydroxy fatty acids				
C_12:0_ 3-OH	tr	tr	0.8	0.8
iso-C_14:0_ 3-OH	4.0	3.7	1.2	tr
C_16:0_ 3-OH	0.7	tr	3.3	–
C_17:0_ 3-OH	0.8	–	–	–
*Summed features				
Sum In Feature 2	tr	0.7	1.8	3.0
Sum In Feature 3	–	tr	–	9.0

Strain: 1, Y139^T^; 2, *Luteolibacter arcticus* CCTCC AB 2014275^T^; 3 *Luteolibacter flavescens* MCCC 1K03193^T^; 4 *Luteolibacter pohnpeiensis* CCTCC AB 2011006^T^. Fatty acids that represented > 10.0% are indicated in bold type. Fatty acids that represent < 1.0% in all columns are omitted. tr, Trace (<0.5%); −, not detected. Summed feature 2 contains C_14:0_ 3-OH and/or iso-C_16:1_; summed feature 3 contains C_16:1_ ω7c and/or iso-C_15:0_ 2-OH.

### 3.3 16S rRNA gene sequence and phylogenetic analysis

**Figure 1:**
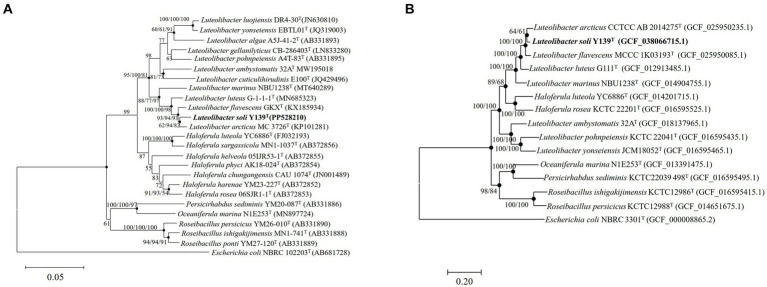
The phylogenetic tree of strain Y139^T^ and other related species, *Escherichia coli* NBRC 102203^T^ was used as the outgroup. **(A)** Neighbor-joining phylogenetic tree based on 16S rRNA gene sequences of strain Y139^T^ and other closely related species. Filled circles indicate branches that were recovered with neighbor-joining, maximum-likelihood, and minimum-evolution methods. Bootstrap values above 50% (1,000 replicates) are shown at branch nodes (NJ/ML/MP). Bar: 0.05 substitutions per nucleotide position; **(B)** The FastTree is based on 120 ubiquitous single-copy proteins. Bootstrap values above 50% (1,000 replicates) are shown at branch nodes. Filled circles indicate that the same topology is also obtained using the IQ-Tree algorithm. *Escherichia coli* NBRC 102203^T^ was used as the outgroup. Bar: 0.20 substitutions per nucleotide position.

To align the 16S rRNA gene sequence of strain Y139^T^ (1,518 bp), the EzBioCloud database was utilized. The sequence similarity to the genus *Luteolibacter* ranged from 92.1 to 98.3%. The results showed that strain Y139^T^ was affiliated with the genus *Luteolibacter* and closely related to *L. flavescens* MCCC 1K03193^T^ and *L. arcticus* CCTCC AB 2014275^T^ with 98.3 and 98.2% 16S rRNA gene similarities, respectively. This is below the threshold for distinguishing between two species (98.6%) (Kim et al., 2014). To explore its evolutionary position, phylogenetic tree based on 16S rRNA gene sequences was constructed. The result shows that strain Y139^T^ was most closely related to *L. arcticus* CCTCC AB 2014275^T^, and should be classified within the genus *Luteolibacter* (Figure 1A). Similar topologies were also obtained with three algorithms (NJ, ML and MP). In addition, the phylogenomic tree reconstructed using protein-coding genes can also lead to the same conclusions. (Figure 1B).

### 3.4 Genomic characteristics

**Figure 2:**
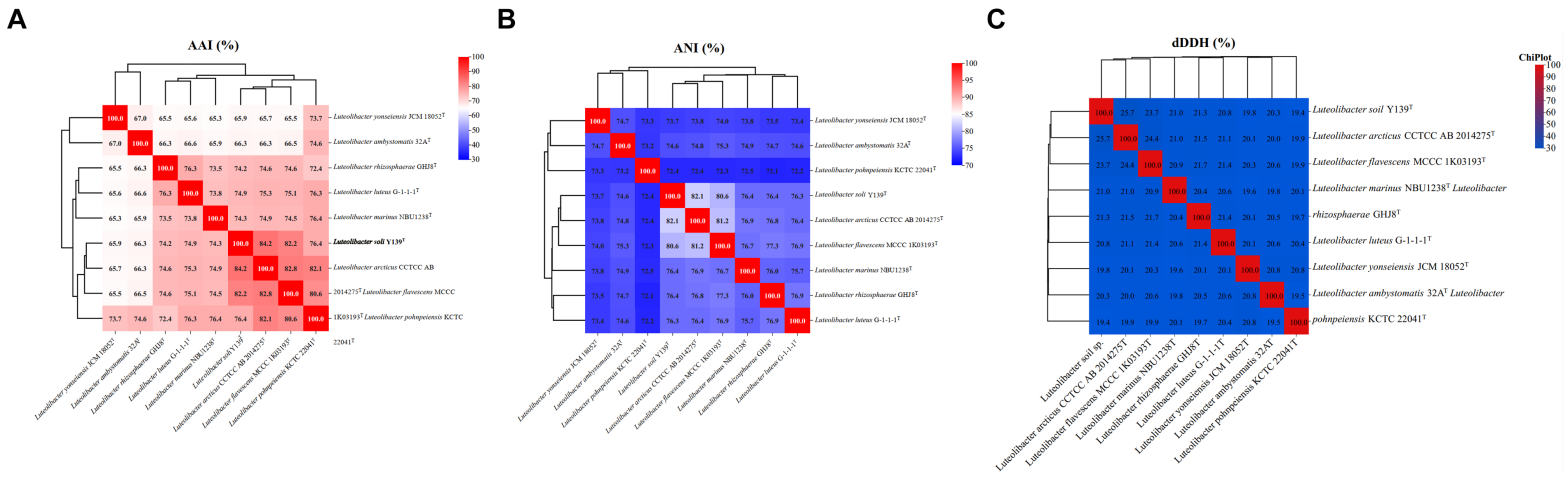
Genomic similarities of strain Y139^T^ to some members of the genus *Luteolibacter*. **(A)** The AAI values between isolates of the genus; **(B)** The ANI values between isolates of the genus; **(C)** The dDDH values between isolates of the genus.

For strain Y139^T^, the draft genome measured 7,106,054 bp in length and consisted of 31 scaffolds, with a genomic DNA G + C content of 62.5%, consistent with the range of other species in the genus *Luteolibacter* (Yoon et al., 2008; Xie et al., 2022). The results of the genome analysis showed a completeness of 98.8%. The draft genome of strain Y139^T^ contains 5,715 genes, which consist of 5,651 protein-coding genes, 5 pseudogenes, and 59 RNA genes (3 rRNA genes, 53 tRNA genes, and 3 non-coding RNA genes). The antiSMASH analysis revealed biosynthetic gene clusters responsible for secondary metabolites such as betalactone, terpenes, type I polyketide synthase (PKS), type III polyketide synthase (PKS), and non-ribosomal peptide synthetase (NRPS) (Supplementary Figure S3). The AAI values were 82.2 and 82.4% between strain Y139^T^ and *L. flavescens* MCCC 1K03193^T^ and *L. arcticus* CCTCC AB 2014275^T^, respectively, exceeding the 62–72% threshold for genus delimitation (Nicholson et al., 2020). The ANI values were 80.6 and 82.1% for strain Y139^T^ compared to *L. flavescens* MCCC 1K03193^T^ and *L. arcticus* CCTCC AB 2014275^T^, respectively, which are below the 95% threshold for species delimitation (Jain et al., 2018). The dDDH values were 23.7 and 25.5% between strain Y139^T^ and *L. flavescens* MCCC 1K03193^T^ and *L. arcticus* CCTCC AB 2014275^T^, respectively, these values are all below the 70% threshold, which is commonly used to delineate a novel species (Goris et al., 2007; Figure 2). These findings indicate that strain Y139^T^ should be a novel species within the genus *Luteolibacter*.

### 3.5 Comparative genomic analysis

In the genus *Luteolibacter*, including strain Y139^T^, genomes ranged from 4,667,805 to 7,486,770 bp, with the genomic DNA G + C content varying from 53.5 to 65.0% (Supplementary Table S1). Analysis of the pan-genome was conducted using orthologous protein groups, showing that the average number was 4,882, with a total of 1,177 core genes accounting for 24% of the genomes in the genus *Luteolibacter* (Supplementary Figure S4A). Each genome of *Luteolibacter* has 23.3–55.2% accessory genes and 21.9–46.7% unique genes (Supplementary Figure S4B). Unique genes exhibited a broader distribution across various metabolic pathways including carbohydrate metabolism, energy metabolism, amino acid metabolism, signal transduction and cellular community (Supplementary Figure S5).

**Figure 3:**
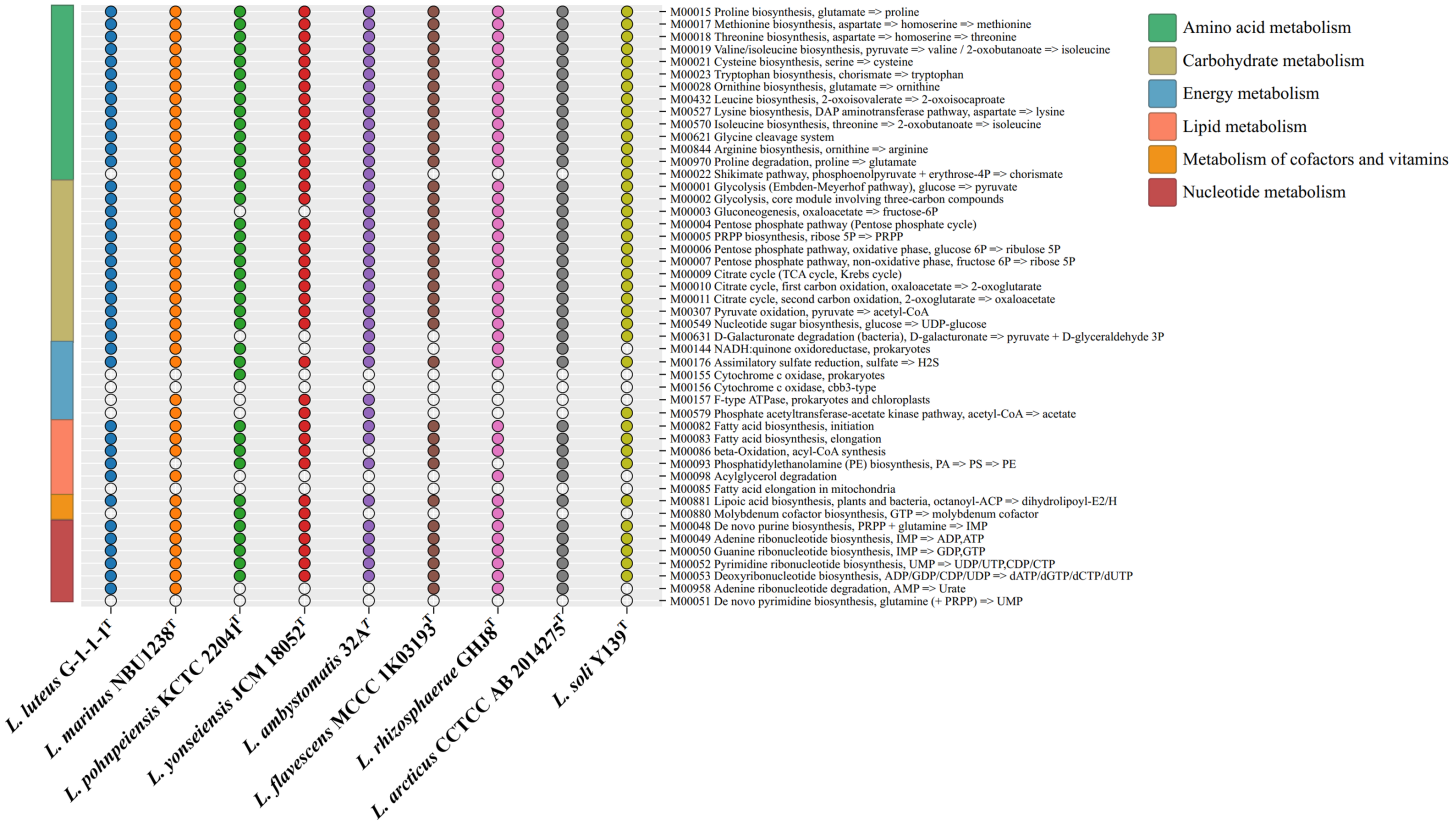
The metabolic module integrity of the genus *Luteolibacter*. The solid circles and hollow circles indicate that the metabolic pathways were complete and incomplete, respectively.

The KEGG distribution analysis of the genus *Luteolibacter* species revealed the significant role of core genes in fundamental metabolic pathways crucial for sustaining life, such as amino acid metabolism, carbohydrate metabolism, and translation. The completion of key pathways like the TCA cycle pathway (M00009), glycolysis pathway (M00001), and pentose phosphate pathway (M00004) was observed, while cytochrome c oxidase (M00156) was not complete (Figure 3). The genus *Luteolibacter* species demonstrated conservatism in carbohydrate metabolism, amino acid metabolism and nucleotide metabolism, with notable variations observed primarily in energy metabolism. All strains within the *Luteolibacter* genus exhibited completeness in assimilatory sulfate reduction (M00176). Most amino acids biosynthesis pathways were complete, including proline biosynthesis (M00015), methionine biosynthesis (M00017), threonine biosynthesis (M00018), valine/isoleucine biosynthesis (M00019), cysteine biosynthesis (M00021), tryptophan biosynthesis (M00023), ornithine biosynthesis (M00028), leucine biosynthesis (M00432), lysine biosynthesis (M00527), isoleucine biosynthesis (M00570) and arginine biosynthesis (M00844) in nine members of the genus *Luteolibacter*. Notably, proline has been identified to enhance bacterial growth under stress conditions, particularly aiding in osmotic stress tolerance through cellular accumulation of amino acids. The survival and growth of bacteria are influenced by their capacity to regulate osmotic pressure by intracellular amino acid accumulation. Based on the RAST genome annotation, gene distribution associated with subsystems falls into 24 different categories, as shown in Supplementary Figure S6.

### 3.6 Biogeographic distribution

**Figure 4:**
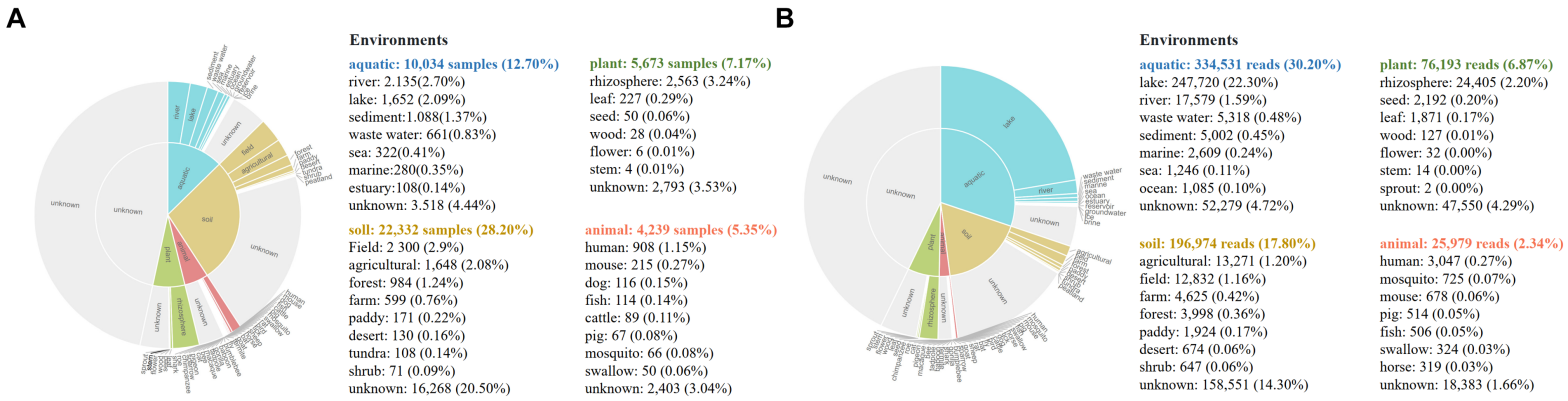
Biogeographic distribution analysis of genus *Luteolibacte*. **(A)** Frequency of samples with representative OTU sequence, by habitat and sub-habitat; **(B)** Abundance of sequencing reads mapping to the representative OTU sequence, by habitat and sub-habitat.

The MAP database^10^ was used to determine the worldwide distribution of the genus *Luteolibacter*. A total of 79,176 samples from 8,000 projects were analyzed to identify the representative sequence. The results of the biogeographic distribution analysis indicated that *Luteolibacter* is widespread across different habitats, including aquatic, soil, animal, and plant environments. Specifically, *Luteolibacter* bacteria were found in 22,332 soil samples (28.2%), 10,034 aquatic samples (12.7%), 5,673 plant samples (7.2%), and 4,239 animal samples (5.4%). Among the known environments, the primary habitats for *Luteolibacter* were identified as the rhizosphere (3.2%), field (2.9%), agricultural areas (2.1%), river (2.7%) and lake ecosystems (2.1%) (Figure 4A). The mapping of database sequencing reads to the standard OTU sequence showed that the lake environment had a predominant proportion (22.3%) of reads from the genus *Luteolibacter* (Figure 4B).

### 3.7 Impact of climate change on genus *Luteolibacter*

**Figure 5:**
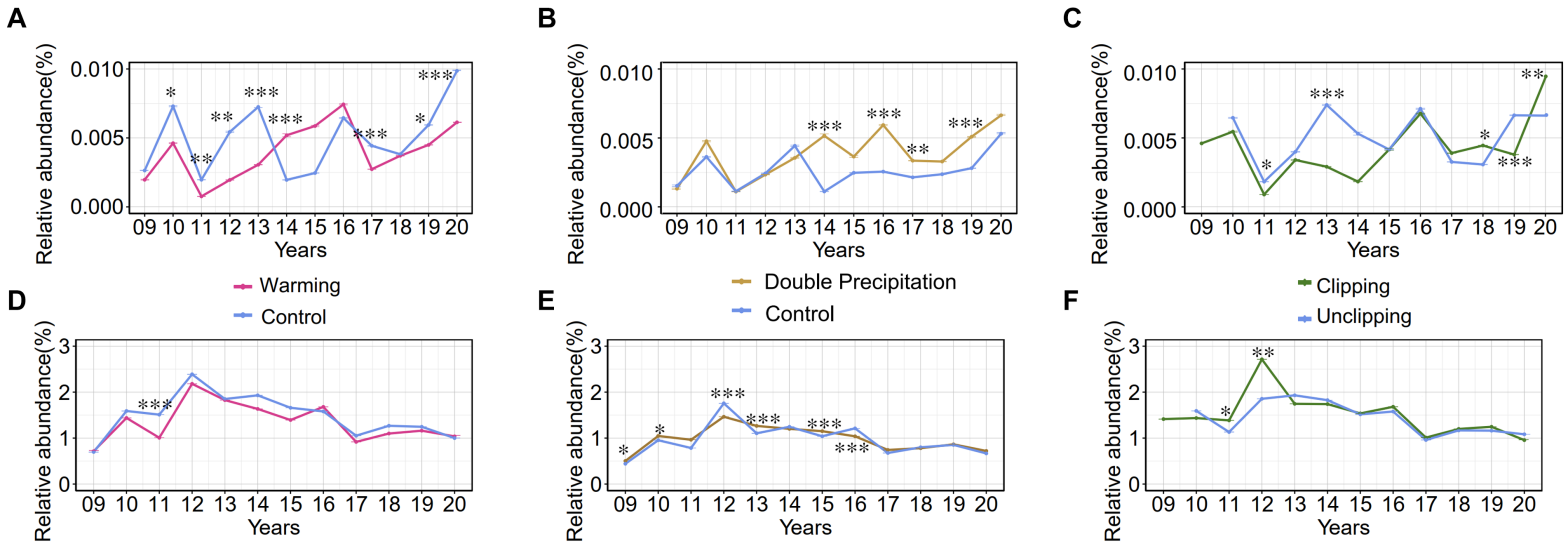
The relative abundance (%) trends of phylum *Verrucomicrobia* and genus *Luteolibacter* from 2009 to 2020 under various conditions. 09 to 20 represent the years 2009–2020, respectively. **(A–C)** Effect of warming/precipitation/clipping on the relative abundance of genus *Luteolibacter*; **(D-F)** Effect of warming/precipitation/clipping on the relative abundance of phylum *Verrucomicrobia*. Data are presented as mean ± SD of the estimated effect sizes. Statistical significance is based on t test, asterisks indicate statistical significance, with the number of asterisks representing the level of significance (* for *p* < 0.05; ** for *p* < 0.01; *** for *p* < 0.001).

The genus *Luteolibacter*, belonging to the phylum *Verrucomicrobiota*, is widely distributed in soil environments. Relative abundances were calculated at the amplicon sequence variant (ASV) level, revealing that the relative abundances of the phylum *Verrucomicrobiota* and the genus *Luteolibacter* in soil samples ranged from 0.3 to 3.3% and from 0 to 0.1%, respectively, from 2009 and 2020. The relative abundances of the genus *Luteolibacter* and the phylum *Verrucomicrobiota* were tracked over several years under three climate change factors: warming, precipitation, and clipping. The genus *Luteolibacter* exhibited higher relative abundances under conditions of increased temperature and double precipitation over time, suggesting that these climate change factors have a substantial impact on its population. Clipping also influenced the relative abundance of the genus *Luteolibacter*, but the effect was irregular and fluctuating (Figures 5A–C). This consistent increase in relative abundance under specific conditions highlights the potential resilience and adaptability of *Luteolibacter* to changing environmental parameters. In contrast, we did not observe similar changes in the phylum *Verrucomicrobiota*. Precipitation had a greater effect on the phylum *Verrucomicrobiota* than warming and clipping, but this effect was variable and fluctuated over time (Figures 5D–F).

## 4 Description of *Luteolibacter soli* sp. nov

### 4.1 *Luteolibacter soli* (so′li. L. gen. Neut. n. *soli* of soil)

Cells of strain Y139^T^ are Gram-stain-negative, non-motile, aerobic, rod-shaped, with the width of 0.4–0.7 μm and the length of 1.5–2.0 μm . They exhibit growth between 20 and 37°C (optimum, 30°C), with pH tolerance from 5.5 to 9.5 (optimum, pH 7.0), and 0–1.0% NaCl concentration (optimum, 0%). The cells display oxidase and catalase activity and can hydrolyze cellulose and starch, but not alginate, casein, or Tweens 20, 40, 60, and 80. The only respiratory quinone is menaquinone-9 (MK-9). The dominant fatty acids are C_16:0_, iso-C_14:0,_ and C_16:1_ *ω*9*c*. The major polar lipids include phosphatidylethanolamine (PE), phosphatidylglycerol (PG), phosphatidyldimethylethanolamine (PME), diphosphatidylglycerol (DPG), and an unidentified lipid (L). Cells test positive for *β-*galactosidase, esterase lipase (C8), esterase (C4), acid phosphatase, naphthol-AS-BI-phosphohydrolase, leucine arylamidase and alkaline phosphatase. In carbon source oxidation tests, negative results are obtained for fusidic acid, *ρ*-hydroxy-phenylacetic acid, d-malic acid, l-malic acid, *γ*-amino-butyric acid, *α*-hydroxy-butyric acid, *α*-keto-butyric acid, *β*-hydroxy-d, l-butyric acid and propionic acid. Cells are also positive for the utilization of tryptophan, sucrose, maltose and l-arabinose. Acid is produced from methyl-*α*-d-mannopyranoside, methyl-*α*-d-glucopyranoside, d-melezitose, glycerol, d-xylose, d-galactose, d-glucose, d-fructose, d-mannose, d-sorbitol, amygdalin, salicin, arbutin, d-cellobiose, d-maltose, d-lactose, d-melibiose, d-saccharose, d-trehalose, inulin, d-raffinose, amidon, esculin ferric citrate, glycogen, gentiobiose, d-turanose, potassium 5-ketogluconate, and potassium gluconate.

Type strain Y139^T^ (= KCTC 92029^T^ = MCCC 1H00491^T^) was isolated from a soil sample in McClain County, Oklahoma, United States. GenBank numbers for the 16S rRNA gene sequence and draft genome of *Luteolibacter* Y139^T^ are PP528210 and JBBUKT000000000.1, respectively.

## Data Availability

The datasets presented in this study can be found in online repositories. The names of the repository/repositories and accession number(s) can be found in the article/Supplementary material.

## Footnotes

^1^https://www.bacterio.net/, 27 March 2024.

^2^ https://blast.ncbi.nlm.nih.gov/Blast.cgi

^3^ https://www.ezbiocloud.net/

^4^ https://www.ezbiocloud.net/tools/ani

^5^ https://ggdc.dsmz.de/distcalc2.php

^6^ https://www.genome.jp/kegg/

^7^ https://rast.nmpdr.org/

^8^ https://antismash.secondarymetabolites.org

^9^ https://microbeatlas.org/

^10^ https://microbeatlas.org/
