# Supplementary material for: Analysis of genomic and characterization features of Luteolibacter soli sp. nov., isolated from soil
Source: Front Microbiol. 2024 Sep 13;15:1483195. doi: 10.3389/fmicb.2024.1483195 (PMC11427321; doi:10.3389/fmicb.2024.1483195)
Supplement: Supplementary file 1 [file Image_1.pdf]

Supplementary Material

Analysis of genomic and characterization features of *Luteolibacter soli* sp. nov., isolated from soil

Jing An[1], Xiaoqi Xuan[1], Yanan Wang[1], Linwei Wu[2,3], Jizhong Zhou[3,4,5,6*] and Dashuai Mu[1,3,7,8*]

[1] Marine College, Shandong University, Weihai, Shandong, China.
[2] Institute of Ecology, Key Laboratory for Earth Surface Processes of the Ministry of Education, College of Urban and Environmental Sciences, Peking University, Beijing, China.
[3] Department of Microbiology and Plant Biology, University of Oklahoma, Norman, OK, USA.
[4] Institute for Environmental Genomics, University of Oklahoma, Norman, OK, USA
[5] Department of Microbiology and Plant Biology, University of Oklahoma, Norman, OK, USA.
[6] State Key Joint Laboratory of Environment Simulation and Pollution Control, School of Environment, Tsinghua University, Beijing, China.
[7] School of Civil Engineering and Environmental Sciences, University of Oklahoma, Norman, OK, USA.
[8] School Key Laboratory of Microbial Technology, Shandong University, Qingdao, Shandong, China.
[9] Weihai Research Institute of Industrial Technology of Shandong University, Weihai, China.

***Correspondence:**
Da-Shuai Mu, Email: dashuai.mu@sdu.edu.cn
Ji-Zhong Zhou, Email: jzhou@ou.edu

Figure S1. Scanning electron micrograph of strain Y139^{T} after growing on R2A plates at 30℃ for 2 days. Bar: 4 μm.

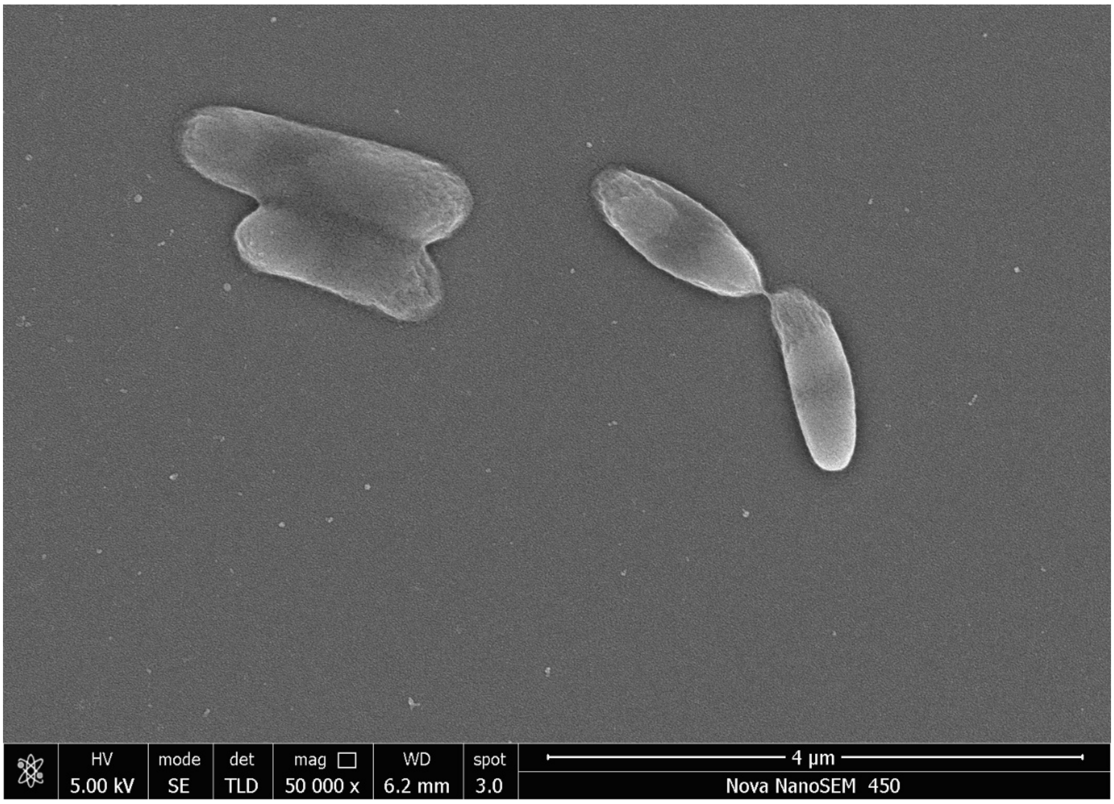

Figure S2. Two-dimensional TLC plate images of polar lipids of strain Y139T (A); *L.arcticus* CCTCC AB 2014275T (B); *L.flavescens* MCCC 1K03193T (C); *L.pohnpeiensis* CCTCC AB 2011006T (D).

Abbreviation: PE, phosphatidylethanolamine; PG, phosphatidylglycerol; PME, phosphatidyldimethylethanolamine; DPG, diphosphatidylglycerol; GL, (unidentified) glycolipid; L, unidentified lipids.

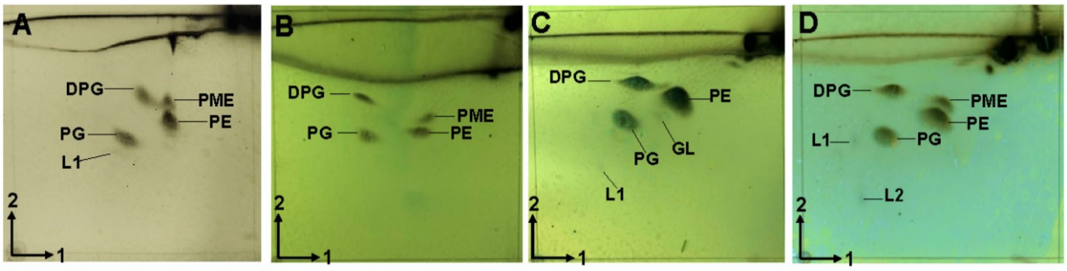

Figure S3. The secondary metabolite biosynthetic gene clusters of all strains in the genus *Luteolibacter*. Solid circles and hollow circles represent the presence or absence of the metabolite, respectively.

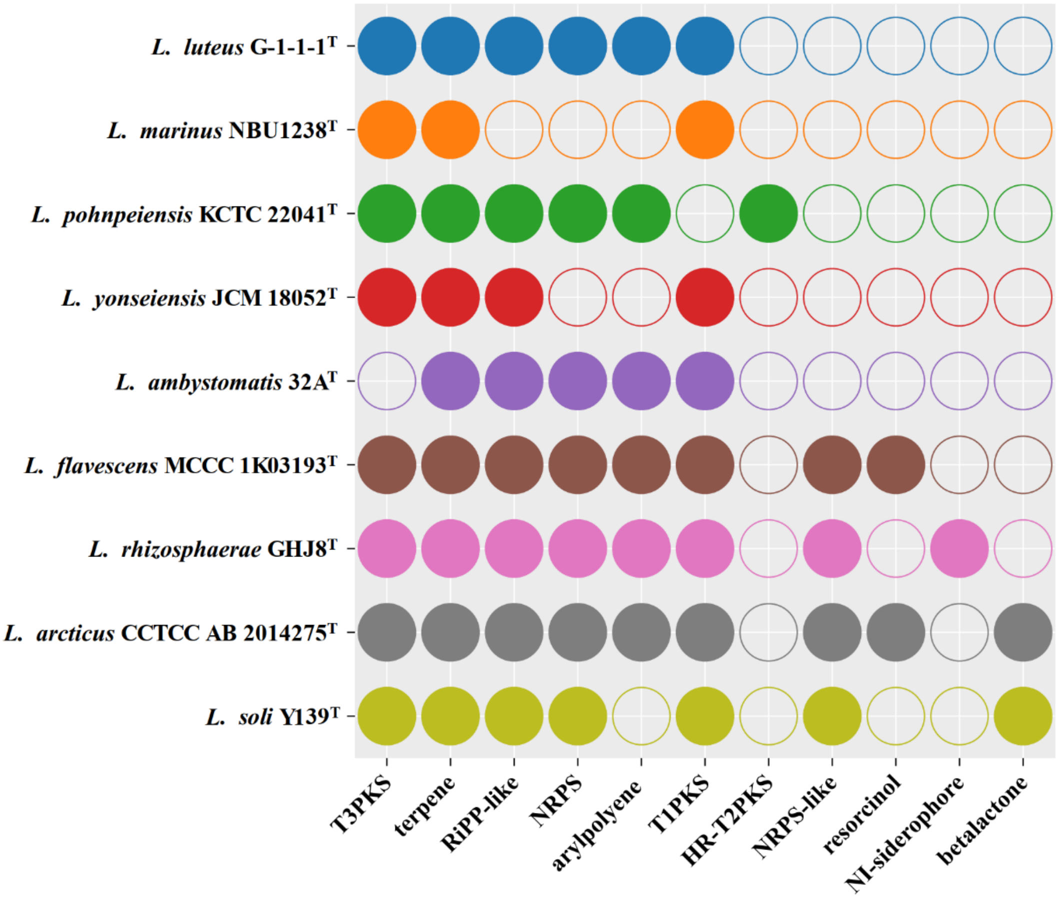

Figure S4. Genome analysis of the genus *Luteolibacter* (9 genomes). (A) Venn diagram displaying the numbers of core gene families and unique genes for each *Luteolibacter* strain. (B) Percentage of core, accessory, and unique genes in each genome.

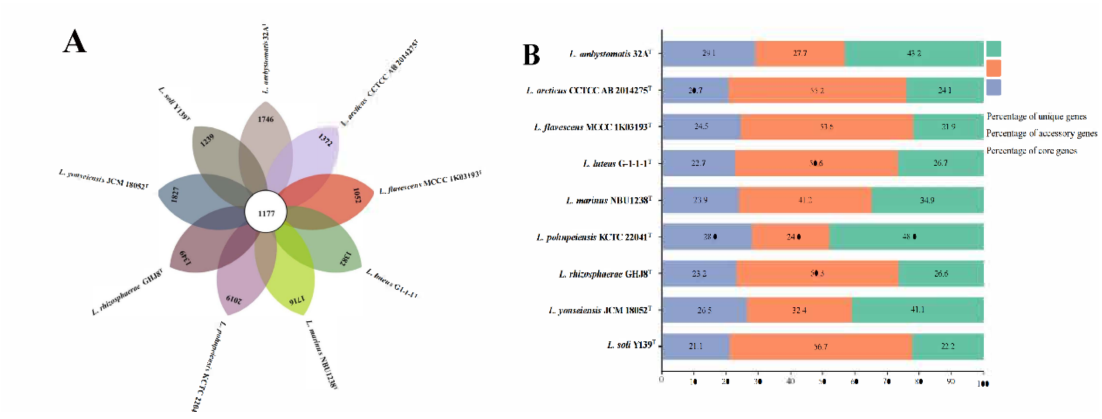

Figure S5: The distribution of core genes, accessory genes and unique genes to different metabolic pathways in the genus *Luteolibacter*.

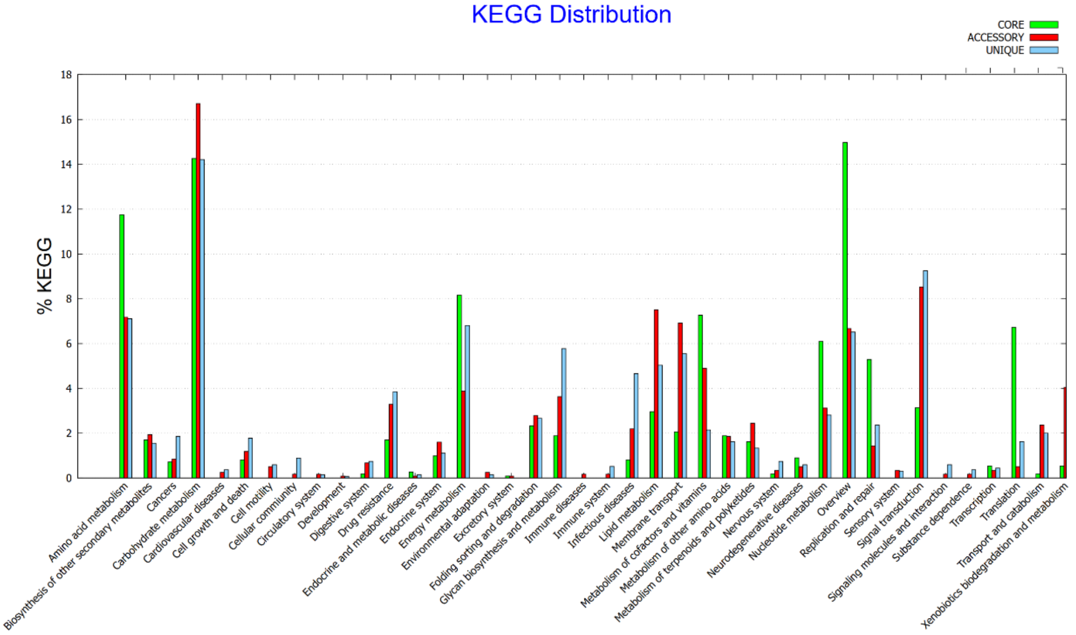

Figure S6: Genes associated with subsystems and their distribution across various categories of strain Y139^{T}.

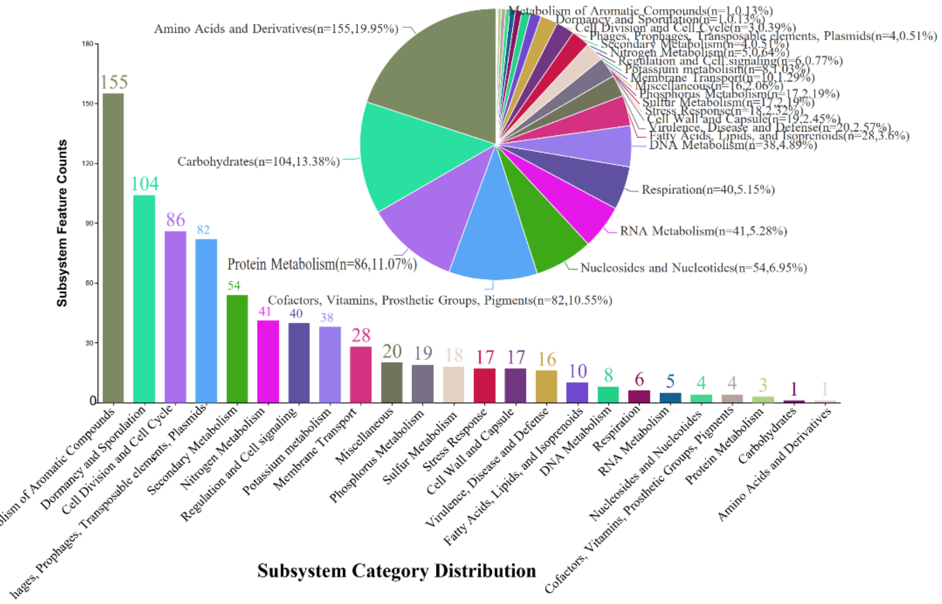

Table S1. Genomic dataset of Y139^T and other strains of genus *Luteolibacter* analyzed in the pan-genome analysis.

Strain	Genome size (Mb)	Number of scaffolds	Completeness (%)	G+C content (%)	Genes	Protein-coding gene	Pseudogene	RNA gene	GenBank
L. ambystomatis 32A^T	5.3	1	99.1	62.0	4178	4112	7	59	GCA_018137965.1
L. arcticus CCTCC AB 2014275^T.	7.5	43	98.5	63.0	5848	5756	31	61	GCA_025950235.1
L. flavescens MCCC 1K03193^T	5.9	73	98.0	64.0	4911	4836	21	54	GCA_025950085.1
L. luteus G-1-1-1^T	6.4	1	98.9	61.0	5268	5176	32	60	GCA_012913485.1
L. marinus NBU1238^T	6.2	355	96.4	65.0	5053	4889	103	61	GCA_014904755.1
L. pohnpeiensis CCTCC AB 2011006^T	4.7	254	97.5	53.5	4251	4180	16	54	GCA_016595435.1
L. rhizosphaerae GHJ8^T	6.2	33	99.0	62.5	5267	5194	18	55	GCA_025950095.1
L. yonseiensis JCM 18052^T	5.7	13	98.6	60.5	4581	4499	9	73	GCA_016595465.1
L. soli Y139^T	7.1	31	98.9	62.5	5715	5651	5	59	GCA_038066715.1
